# Reminiscences of the Insane

**Published:** 1887-01-29

**Authors:** 


### Reminiscences of the Insane.

By a Mental Physician.

(*Continued from page 254.*)

Facial Expression of Patients.

Among the Reminiscences of the Insane none are more distinctive, and none more tenaciously retained in the memory, than the features and expression of patients. There is the physiognomy and pathognomy ; the former by no means necessarily indicating mental disease, although sometimes suggestive of a constitutional predisposition to mental derangement. Hence it is allowable to speak of an insane temperament. And, of course, among idiots and imbeciles the native defect is written in sufficiently broad characters upon the face and brow, and indeed the whole body. More frequently, however, there are the temporary changes in the facial expression which constitute pathognomy. In the long portrait-gallery which arises before the mental eye of the physician who has lived with the insane, many peculiarities of look and gaze group themselves into more or less distinct classes.

Acute Mania.

There arises the wild and hopelessly lost expression of the ravingly mad patient, with rapid alternations of excitement and vacant abstraction. If the popular idea of a maniac is exaggerated by the impression received from the typical madman of a period when the chains by which he was secured, and the brutal treatment to which he was subjected, intensified instead of soothing the frenzy of the lunatic, it is still easy enough, unfortunately, to see an acute attack of mania in which every feature and every muscle are in a state of intensest excitement, the patient presenting a picture which cannot be said to fall short of the most violent Bedlamite of a former generation. There have always been, and still are, patients possessed with the one delight of destruction in every possible direction, to control which by forcible means may be alike injurious and cruel, while to allow the expenditure of this excessive force and excitement to find its outlet in the open air without any unnecessary limitation, affords a safety-valve by which it may escape. We have known patients of this class describe their feelings after recovery as acutely pleasurable, although in many instances the memory of the delirium through which they have passed, is so confused that little can be gleaned from their recollections, and sometimes there is an absolute blank. It is, at any rate, some satisfaction to know that in either case there has not been that mental suffering which would result from a maniac being able to contrast his state of ungovernable fury with his healthy condition.

Melancholia.

From this portrait the physician's retrospective glance turns to the striking contrast of the melancholy patient, whose physiognomy presents an intensification of that with which everyone is familiar as a depressed look indicative of misery. So characteristic of the melancholy mood is the constriction of the eyebrows and wrinkling of the forehead, that the term "grief muscles" has become quite familiar. The mental state may be one of great activity,—for there is no inconsistency in speaking of acute or even excited depression ; and here compressed lips and concentration of all the features point to the hideous idea which dominates the mind, and holds the unhappy patient in its iron grasp. On the other hand, there is the passive form of melancholia, with open mouth and relaxed features, which tells of a despair which has ended in utter indifference, and which bears the same relation to the acute variety as paralysis does to spasm.

Suffering often Real; may be Apparent.

That there is frightful mental suffering in melancholia, no one who has listened to the description given by patients labouring under it, whether during the attack or after it can doubt; at the same time there are cases in which the look of woe and the constant moaning are not true indications, one is glad to think, of the patient's torment, but have become to a large extent mechanical and automatic. A certain habit has become established, and this persists after the acute feeling has to a very considerable degree subsided. In the really acute cases a patient will say, and no doubt with perfect truth, that the depth of their sense of misery is indescribable, and that they only wish that their physician could experience it for one hour, and thus know from personal feeling how intense is their feeling of despair. We have heard a clergyman in an asylum speak in terms which conveyed the idea of the most terrible obsession, suffering beyond which it would be impossible to conceive in store for the wickedest and most impenitent of men. Strange to say, this clergyman, possessed as he was with the most horrible delusions of everlasting perdition, was able to control himself sufficiently to read the lessons during Divine Service in the chapel of the asylum.

Exalted Delusions.

Then there are patients labouring under neither acute mania nor melancholia, whose physiognomy is a faithful tell-tale of their thoughts and feelings. Possessed by an exalted delusion, a patient may carry in his face the signs of happy schemes and world-wide plans, springing from the brain which has lost its balance and can no longer condescend to subject the outcomes of sentiment and benevolent feeling to the dry tests demanded by reason and the cold requirements of logic. The whole expression is lit up with joyous self satisfaction, and marks in clearest lineament the immovable certainty of the hope by which he is animated. Everything is converted into gold, and there is no limit to the beautiful presents of money unhesitatingly made by such a patient to the doctor or his attendant. Gaiety of heart, the sublime disregard of every suggested difficulty, the rapid passage from one optimistic delusion to another, the rapid succession of fresh creations of the patient's exalted fancy, mark this class of happy lunatics, and present a pitiful picture of human erring,

Satire on Human Folly.

and a satire on the oft-repeated schemes of wild reformers outside asylums, some of whom, in truth, have an equal claim on the sheltering care of a hospital. How many bubbles would have broken before the infliction of wide-spread disappointment and loss of money—causing untold misery to the community at large—had such well-intentioned but crack-brained enthusiasts been placed under judicious medical care !

Again, a patient may be haunted by delusions which

Delusions of Suspicion.

do not impart the expression of mania or melancholy, and possess none of the sanguine features just described. He may be intensely suspicious, and his physiognomy present all the marks of this mental condition. He sees in everything that happens a fresh proof of his suspicious misgivings; the ticking of the clock on the wall forms intelligent words and sentences. In fact although a new language is not formed, inanimate objects—even plants and trees—speak, like Balaam's ass, what to them is a new language, and in a somewhat different sense than that intended by the poet—there are sermons in stones and brooks; while everything which surrounds him in the diseased world inhabited by the spectres born of his delusions becomes vocal, and full of the sounds created by the morbid vibrations of his own nervous system. A patient, after his recovery, thus describes his own interpretation of what passed daily around him.

The Inorganic World Speaks.

Patient describes his own Feelings.

"My chief impression was, that, wherever I happened to be, my thoughts were known to most other persons, which, together with the remarks I heard, caused me much misery. At first I believed myself to have been the subject of a process of mesmerism or hypnotism, by means of a sort of conspiracy against me to make me commit some foolish or rash act; but although I was reduced to almost a mad condition, I suppressed my anger and excitement, and tried to let the attack wear itself out, by remaining passive under insults and provocation. . . . When I found myself in possession of an influence over others by reason of all my thoughts being known to them, my first endeavour was to try to lead others into a way of thinking of their own safety and salvation, and I seemed for a time to have considerable success; but then a reaction took place, and I began to hear words of a very blasphemous kind when dogs barked; the crowing of cocks also took the form of words, as did the cawing of rooks or crows; the chirping of sparrows; the puffing of railway engines; the working of machinery; the ringing of bells; the ticking of clocks; the scratching of a pen; sometimes the splashing of water when it was poured out; the falling together of cinders in the stove. On some occasions I heard whisperings from plants asking for water, both in a greenhouse and in the garden. I heard whisperings when alone and when sitting with friends. I even heard voices close to my ear as distinctly as if by a telephone (when my head has been covered over by the bedclothes), and I have heard voices and sounds proceeding from the sky or heavens."

This patient has never been inclined to believe in modern Spiritualism, nor was he superstitious. He felt, however, as if he was a sort of medium, so that other persons' minds were influenced by his, and that the good purpose to which he endeavoured to turn such an influence was continally meeting with opposing influences, in the form of evil together sounds, with what he speaks of as "most unaccountably wicked thoughts which flashed like fiery darts through my mind."

---

THE plans of the new Children's Hospital at Gateshead are now under the consideration of the committee. The foundation stone will probably be laid during the present year, so that the Gateshead Children's Hospital will be another memorial of Her Majesty's Jubilee.

